# Supplementary material for: Direct liquid transmission of sound has little impact on fermentation performance in Saccharomyces cerevisiae
Source: PLoS One. 2023 Feb 17;18(2):e0281762. doi: 10.1371/journal.pone.0281762 (PMC9937469; doi:10.1371/journal.pone.0281762)
Supplement: S1 Appendix — (DOCX) [file pone.0281762.s002.docx]

S1 APPENDIX

**MATLAB bespoke code:**

filename='';

[dat,fs]=audioread(filename);

dat=dat(:,1);

n=1;

samr=fs; % sampling rate

nstart=n*samr; % starting point for plot/spectrum

nsam=fs; % number of samples

tlo=4; tup=15; % time window for plot - NB seconds

nlo=nstart+nsam*tlo+1; % first data sample index

nup=nstart+nsam*tup; % last data sample index

data=data(nlo:nup)'; % data segment for plot/spectrum

data=data*109685697.2; % apply calibration

npts=length(data); % number of points in data segment

tint=npts/samr; % time interval of data segment

t=tlo+(1:npts)/samr; % time axis for plot

subplot(411);

plot(t,data);

%{

flo=20; fhi=22000;

fdata=bpfilt(data,flo,fhi);

subplot(412);

plot(t,fdata);

%}

subplot(413);

pfdata=abs(data).^2; % is Prms^2 of filtered data

smp=smooth(pfdata,5);

dbpd=10*log10(smp); % converts into dB

plot(t,dbpd);

dbav=10*log10(sum(pfdata)/length(pfdata));

%axis([min(t),max(t),min(dbpd),max(dbpd)]);

spec=fft(data)/npts; % fft of data segment NB Matlab fft drops 1/N

fnc=1/tint; % frequency increment in spectrum

nfreq=floor(npts/2); % number of frequencies in spectrum

f=fnc*(0:nfreq-1)/1000; % frequencies for plot - /1000 for kHz

pspec=abs(spec(1:nfreq)).^2; % power spectrum

pspec=pspec/fnc;

dbspec=10*log10(pspec);

subplot(414); % spectrum of filtered data

smdbspec=10*log10(smooth(pspec,10));

semilogx(f,smdbspec);

axis([0.1,24,min(smdbspec),max(smdbspec)]);

%axis([0.1,24,-120,-20]);

title(['Mean level = ',num2str(dbav)]);

figure(gcf)
