# Supplementary material for: Direct liquid transmission of sound has little impact on fermentation performance in Saccharomyces cerevisiae
Source: PLoS One. 2023 Feb 17;18(2):e0281762. doi: 10.1371/journal.pone.0281762 (PMC9937469; doi:10.1371/journal.pone.0281762)
Supplement: Attachment — Submitted filename: Responses to Reviewers.docx [file pone.0281762.s003.docx]

**Responses to Reviewers**

**Editor**

1. Please ensure that your manuscript meets PLOS ONE's style requirements, including those for file naming.

We think we have now met all PLOS ONE’s requirements, but please let us know if we have inadvertently missed any.

2. We note that the grant information you provided in the ‘Funding Information’ and ‘Financial Disclosure’ sections do not match.

When you resubmit, please ensure that you provide the correct grant numbers for the awards you received for your study in the ‘Funding Information’ section.

Oh, that is strange. However, we are not able to find a “Funding Information” section in our submission to check the error. This section is referred to in the “Financial Disclosure” instructions as coming below, but it is not there (in our submission). As far as we can tell, the information in “Financial Disclosure” section is correct. However, if you could list the discrepancy, we can quickly check.

3. Thank you for stating the following in the Acknowledgments Section of your manuscript:

"We thank Sylvie Hermann-Le Denmat (University of Auckland) for technical assistance, and Michael Kingan, Gian Schmid and Andrew Hall (University of Auckland) for kindly providing assistance with the anechoic chamber. This work was supported by a Smart Ideas grant from the New Zealand Ministry for Business, Innovation, and Employment [UOAX1713]."

We note that you have provided funding information that is not currently declared in your Funding Statement. However, funding information should not appear in the Acknowledgments section or other areas of your manuscript. We will only publish funding information present in the Funding Statement section of the online submission form.

Please remove any funding-related text from the manuscript and let us know how you would like to update your Funding Statement. Currently, your Funding Statement reads as follows: "This work was supported by a Smart Ideas grant from the New Zealand Ministry for Business, Innovation, and Employment [UOAX1713; https://www.mbie.govt.nz/science-and-technology/science-and-innovation/funding-information-and-opportunities/investment-funds/endeavour-fund/] to ARDG, AJ, SV-B, PS and GE. he funders had no role in study design, data collection and analysis, decision to publish, or preparation of the manuscript."

Please include your amended statements within your cover letter; we will change the online submission form on your behalf.

We have removed the funding information from the Acknowledgements. The “Funding Statement” text you quote here is the same as the “Financial Disclosure” text as far as we can tell. So, as outlined above, that is correct except for one small typo: If you could please change “.. and GE. he funders..” to read “.. and GE. The funders..”.

4. In your Data Availability statement, you have not specified where the minimal data set underlying the results described in your manuscript can be found. PLOS defines a study's minimal data set as the underlying data used to reach the conclusions drawn in the manuscript and any additional data required to replicate the reported study findings in their entirety. All PLOS journals require that the minimal data set be made fully available.

Upon re-submitting your revised manuscript, please upload your study’s minimal underlying data set as either Supporting Information files or to a stable, public repository and include the relevant URLs, DOIs, or accession numbers within your revised cover letter.

We will update your Data Availability statement to reflect the information you provide in your cover letter.

Thanks for pointing this out. We made a Supporting Table that contains all the raw data that are not included in main Tables, as well as a Supporting Appendix, but neglected to cite these in the manuscript. This has now been rectified, and we have also added more details to the Supporting Table to make the nature of the data more clear. We do not believe our Data Availability statement requires amending (aside from a minor wording correction), as it correctly lists where the data can now be found, although we are happy to take your advice if you think it should be changed. For clarity, the data are available through main manuscript Tables, Supporting information files, and a Figshare repository listed in the Data Availability statement.

5. Please include captions for your Supporting Information files at the end of your manuscript, and update any in-text citations to match accordingly.

We have included captions now as instructed. We believe we have also now named and correctly cited all the supporting information files.

**Reviewer #1:**

Overall, I find the manuscript technically sound with data supporting the conclusions. It is however important to point out that the study is performed with white noise signals and thus the conclusions drawn are valid for this class of signals. Please clarify this in abstract, discussion, conclusions etc.

Yes, excellent point – we have added to all three places as requested.

I would also appreciate a rationale and/or discussion for the choice of white noise signals as stimuli. Many if not most mechanical (including acoustic) stimuli in nature are of periodic nature. For instance, is the studied organism S. cerevisiae reported to oscillate at a frequency around 1 kHz (see e.g. Pelling et al., Science. 2004 Aug 20;305(5687):1147-50). Also, some of the provided references with contradicting results used music signals as stimulary sources.

We have added consideration of these points to the Discussion, which we hope now captures what the Reviewer was intending.

Minor comment: Ergosterol stock solution to growth medium. Please clarify if the ergosterol addition was 12 mL TOTAL or 12 mL per L medium.

Thanks for pointing out this oversight – we have clarified this now.

**Reviewer #2:**

Were the anechoic chamber dimensions similar in control as it was in the test experiments?

This is a good point – we did not make the nature of the anechoic chamber clear enough. The anechoic chamber is a large facility that accommodated the water barrel, and thus the same chamber was used throughout this study. We have amended the text in a number of places to better describe our anechoic chamber setup. Hopefully these changes make the nature of our setup more clear.

Moreover, the material and placement of the fermentation bags/vessels should be same for all control and test experiments. Spatial variations in case of sound waves are known to alter the acoustic intensity and may result to artifacts due to fluctuating intensity (Sutkar and Gogate, 2009, Moholkar et al., 2000; Gogate et al., 2002).

Sutkar, V.S., Gogate, P.R., 2009. Design aspects of sonochemical reactors: techniques for understanding cavitational activity distribution and effect of operating parameters. Chem. Eng. J. 155, 26–36.

Moholkar, V.S., Sable, S.P., Pandit, A.B., 2000. Mapping the cavitation intensity in an ultrasonic bath using the acoustic emission. AIChE J. 46, 684–694.

Gogate, P.R., Tatake, P.A., Kanthale, P.M., Pandit, A.B., 2002. Mapping of sonochemical reactors: review, analysis, and experimental verification. AIChE J. 48, 1542–1560.

We realized, thanks to this comment, that we did not describe our approach with respect to this clearly. Therefore, we have added the following text to the Results section, which we think should allay the reviewer’s potential concerns as well as clearly describing our approach:

“*To remove any biases arising from spatial variation in acoustic intensity (Gogate, Tatake, Kanthale, & Pandit, 2002; Moholkar, Sable, & Pandit, 2000; Sutkar & Gogate, 2009), the placement of fermentations in the barrel was kept constant between experiments, but the position of the fermentation replicates was randomised following each sampling timepoint*.”

Why did author not consider testing the sound beyond the range they have included as the enhancement in ethanol titer has not been found here. There are some interesting observations after mechanistic investigations and more enhancement in the studies where fermentation was performed under intermittent ultrasonic irradiation. Along with their results, authors can discuss the findings in other sound-intensified ethanol fermentation [Singh et al. 2015, Ultrasonics sonochemistry 21 (1), 200-207; Singh et al. 2015, Bioresource Technology 188, 287-294; Sulaiman et al. 2021, Biochemical Engineering Journal 54 (3), 141-150)]

We focused on audible sound because, in our opinion, it has received less consistent attention than ultrasound, for example, which as the Reviewer points out has been reported to induce responses in microorganisms. We have added some text about these points to the Discussion, which we hope is now satisfactory.
